# Genetic interaction effects reveal lipid-metabolic and inflammatory pathways underlying common metabolic disease risks

**DOI:** 10.1186/s12920-018-0373-7

**Published:** 2018-06-20

**Authors:** Hyung Jun Woo, Jaques Reifman

**Affiliations:** Biotechnology High Performance Computing Software Applications Institute, Telemedicine and Advanced Technology Research Center, U.S. Army Medical Research and Materiel Command, Fort Detrick, MD USA

**Keywords:** Metabolic syndrome, Type 2 diabetes, Coronary artery disease, Hypertension, Epistasis, Genome-wide association studies

## Abstract

**Background:**

Common metabolic diseases, including type 2 diabetes, coronary artery disease, and hypertension, arise from disruptions of the body’s metabolic homeostasis, with relatively strong contributions from genetic risk factors and substantial comorbidity with obesity. Although genome-wide association studies have revealed many genomic loci robustly associated with these diseases, biological interpretation of such association is challenging because of the difficulty in mapping single-nucleotide polymorphisms (SNPs) onto the underlying causal genes and pathways. Furthermore, common diseases are typically highly polygenic, and conventional single variant-based association testing does not adequately capture potentially important large-scale interaction effects between multiple genetic factors.

**Methods:**

We analyzed moderately sized case-control data sets for type 2 diabetes, coronary artery disease, and hypertension to characterize the genetic risk factors arising from non-additive, collective interaction effects, using a recently developed algorithm (discrete discriminant analysis). We tested associations of genes and pathways with the disease status while including the cumulative sum of interaction effects between all variants contained in each group.

**Results:**

In contrast to non-interacting SNP mapping, which produced few genome-wide significant loci, our analysis revealed extensive arrays of pathways, many of which are involved in the pathogenesis of these metabolic diseases but have not been directly identified in genetic association studies. They comprised cell stress and apoptotic pathways for insulin-producing β-cells in type 2 diabetes, processes covering different atherosclerotic stages in coronary artery disease, and elements of both type 2 diabetes and coronary artery disease risk factors (cell cycle, apoptosis, and hemostasis) associated with hypertension.

**Conclusions:**

Our results support the view that non-additive interaction effects significantly enhance the level of common metabolic disease associations and modify their genetic architectures and that many of the expected genetic factors behind metabolic disease risks reside in smaller genotyping samples in the form of interacting groups of SNPs.

**Electronic supplementary material:**

The online version of this article (10.1186/s12920-018-0373-7) contains supplementary material, which is available to authorized users.

## Background

A growing proportion of the world population suffers from metabolic diseases, including type 2 diabetes (T2D), coronary artery disease (CAD), and hypertension (HT), many of which co-occur with obesity [1]. Major symptoms of T2D are linked to the loss of control in the body’s insulin-mediated glucose metabolism, leading to hyperglycemia. Deficient insulin secretion by pancreatic β-cells and, to a lesser extent, insulin resistance in peripheral tissues with the resulting burden on normal β-cell function, underlie T2D pathogenesis, which has strong genetic risk factors [2–4]. Cardiovascular diseases, such as CAD and HT, also have significant genetic risk components [5]. In CAD, lipid plaques build up in blood vessels and attract monocytes and induce inflammatory responses, leading to rupture and thrombus formation in atherosclerosis [6]. High blood pressure (or HT) is believed to be closely related to abnormalities in renal salt excretion and vascular tone, affecting body fluid volume and resistance to blood flow, respectively [7].

Significant advances in understanding the genetic basis of the pathogenesis of these common metabolic diseases have been made possible by genome-wide association studies. In T2D, studies characterizing common variants cumulatively led to the discovery of ~ 80 associated loci [8–13], and additional insights have been gained by more recent studies of rare variants [14]. The loci with the strongest associations include those near *TCF7L2* and *CDKAL1* [9]. The specific mechanism by which the *TCF7L2* locus affects T2D susceptibility is under active investigation, including potential roles played by alternative polyadenylation of its intronic regions [15] that can be characterized by high-throughput sequencing [16]. The loci most strongly associated with CAD number up to ~ 50, including 9p21 near *CDKN2A/B* and others [8, 17–22]. Association studies linking variants to blood pressure measurements and HT also identified ~ 50 loci [23–28], with evidence for enrichment of methylated single-nucleotide polymorphisms (SNPs) associated with these traits [29]. Such large-scale meta-analyses, which evaluate most of the genome-wide variants with relatively large minor allele frequencies, offer a powerful means to discover and replicate susceptibility loci without potential biases that could arise when selectively targeting candidate genes or relying on manually curated gene sets.

However, the use of independent SNPs as the unit of genetic factors leads to the ambiguity of the identity of true causal SNPs and genes within a locus in which SNPs are in linkage disequilibrium (LD). Therefore, it is difficult to gain unequivocal biological insights from the list of loci, despite the increasingly large sample sizes and significance levels of associations discovered. Although a conditional analysis can narrow down potential lists of causal SNPs, it assumes that one or a few causal SNPs in a locus underlie the associations of neighboring SNPs in LD. However, many common diseases are highly polygenic, with individual variants contributing only small effects to the overall genetic susceptibility. These polygenic risk factors likely contain non-additive interaction effects, which are not captured by independent loci (IL; i.e., non-interacting SNPs) or pairwise tests.

In this work, we characterized the collective, non-additive, genetic interaction effects associated with three representative metabolic diseases (T2D, CAD, and HT) using a recently developed discrete discriminant analysis (DDA) approach [30]. We employed gene- and pathway-based SNP groups as units of genetic factors, and evaluated their association with the phenotypes while including the net aggregated sum of interaction effects involving all SNPs within the group. In contrast to approaches that test individual SNP pairs separately for association, DDA forgoes pinpointing strongly associated SNPs and variant pairs within a gene- or pathway-group. Instead, it derives statistics that represent the combined additive and interaction effects of the group of SNPs as a whole. Although this approach shares its spirit with other aggregated tests, such as the Sequence Kernel Association Test [31], it provides the unique advantage of including interaction effects. We previously applied this approach to age-related macular degeneration [30], autoimmune diseases [32], and psychiatric disorders [33]. In the latter work, we found arrays of genes and pathways whose association was significant only when interactions were taken into account.

For the three metabolic disease phenotypes, we used the Wellcome Trust Case-Control Consortium (WTCCC) datasets [8] and demonstrated that the collective inference approach described above allows for the discovery of a large array of pathway groups aligned with the expected pathogenesis mechanisms. Comparisons with independent-SNP results and standard enrichment-based gene/pathway analysis support the view that the enhanced association of collectively interacting gene sets is a recurring feature within the genetic architecture of common polygenic diseases. To further support our conclusions and gain additional insights into collective inference outcomes derived from different types of data, we additionally used a recent data set of genome-wide characterizations of outbred mice to support our T2D-associated results.

## Methods

### Genotyping data

We obtained T2D, CAD, and HT datasets from the WTCCC study [8]. We formed case and control data sets based on the quality control reported in the original study to obtain 2938 control individuals (shared) and 1924 (T2D), 1926 (CAD), and 1952 (HT) case individuals. We performed preliminary IL association analyses on quality-controlled SNP data, selected SNPs with independent-SNP *p*-values < 10^−3^, and removed those with poor clustering in raw genotype-call intensity distributions as well as those without associated SNPs in close proximity. This procedure led to 392,615, 392,668, and 392,752 SNPs for T2D, CAD, and HT, respectively.

We used the Carworth Farm White mice genotyping data reported by Nicod et al. [34] and formed control and case groups of T2D by assigning animals with blood glucose levels (“Bioch.Glucose”) higher than 14.0 mmol/L as case individuals. This classification led to 61 case and 1131 control animals. We used all quality-controlled autosomal SNPs and rounded off imputed dosages into integers to obtain 353,697 variants.

### Association scans

**Fig. 1:**
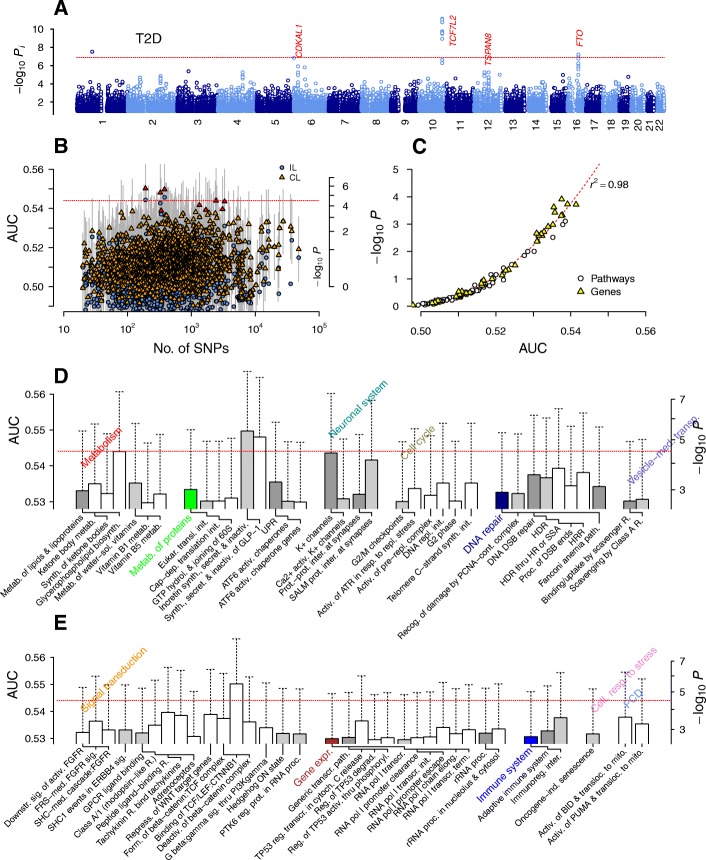
Association test results for type 2 diabetes. Dotted horizontal lines represent the Bonferroni threshold. **a** Non-interacting single-nucleotide polymorphisms (SNPs). **b** Pathways with (collective loci inference; CL) and without interaction effects (independent loci; IL) with varying numbers of SNPs in each group, scored with the cross-validation prediction measure, the area under the curve (AUC). Red symbols represent those with a false discovery rate (FDR) below 0.05. **c** Regression of *p*-values against the AUC (dotted line: fit to quadratic orthogonal polynomial). **d**, **e** Top-ranked pathways organized into the Reactome hierarchy (dendrograms). activ., activates/activation/activated; biosynth., biosynthesis; cont., containing; cytoch., cytochrome; deactiv., deactivation; degrad., degradation; dep., dependent; downstr., downstream; DSB, double-strand break; elong., elongation; eukar., eukaryotic; expr., expression; FGFR, fibroblast growth factor R.; form., formation; GLP, glucagon-like peptide; GPCR, G protein-coupled R.; HDR, homology-directed repair; HR, homologous recombination; HRR, HR repair; hydrol., hydrolysis; inactiv., inactivation; ind., induced; init., initiation; inter., interaction; K+, potassium; med., mediated; metab., metabolism; mito., mitochondria; path., pathway; PCD, programmed cell death; phosphoryl., phosphorylation; pol, polymerase; proc., processing; prot., protein; R., receptor; recog., recognition; reg., regulates; repl., replicative; repress., repression; resp., response; SALM, synaptic adhesion-like molecule; secret., secretion; sig., signaling; sol., soluble; SSA, single-strand annealing; synth., synthesis; term., termination; thru, through; transcr., transcription; transl., translation; transloc., translocation; transp., transport; UPR, unfolded protein response

For all association scans, we used the genotypic model with two degrees of freedom for each SNP and 4 degrees of freedom for each interacting pair. We used the non-interacting special case of DDA for IL analyses shown in Manhattan plots in Figs. 1, 5, and 6. We downloaded the gene-set list of Reactome pathways on December 23, 2016 and considered all pathways excluding those belonging to *Disease* class. We assigned SNPs into genes by proximity (50 kb within the coding region) and formed the union of SNPs for each gene set corresponding to pathways, which led to 1580 pathways containing 20 SNPs or more for each data set. We scored each pathway-based SNP group using the mean-field collective inference of DDA under 5-fold cross-validation, where we maximized the area under the curve (AUC) of the receiver operating characteristic with respect to the regularization parameter ε by varying it between 0 (non-interacting) and 1 (fully interacting). We formed analogous gene-based SNP groups containing one or more unique sets of SNPs, obtaining 16,786, 16,785, and 16,785 SNP sets for T2D, CAD, and HT, respectively, which were scored as for pathways. For outbred mice, we used all mouse Reactome pathways with human counterparts and formed SNP sets based on the corresponding mouse ortholog gene sets. To reduce the high degree of local LD within the mice genotyping data [34], we pruned SNPs corresponding to each pathway prior to inference using PLINK [35] with an LD threshold of 0.9.

### Controlling false positives

We made a selection of genes and pathways containing low numbers of SNPs and repeated collective inferences for each SNP group with phenotype-label permutation. The corresponding *p*-values were then estimated by the fraction of instances for which the AUC was higher than under the alternative hypothesis. We found that optimizing the AUC for each replicate under the null hypothesis with respect to ε led to better agreement of the *p*-value distribution with the expected null distribution (Fig. 4).

## Results

### Type 2 diabetes

We first characterized the genome-wide distribution of the association level of individual SNPs with T2D disease status, using the special case of DDA with interaction effects turned off. The IL *p*-value profile was consistent with the original report [8], showing the strongest association in the *TCF7L2* locus [36], followed by the locus near *FTO* on chromosome 16q, *CDKAL1* on 6p22, and *TSPAN8* on chromosome 12 [37] (Fig. 1a). These loci represent a relatively small subset of all known T2D-associated loci from large meta-analyses [13], reflecting the smaller sample size of the current data set—1924 case individuals in WTCCC versus, e.g., 26,488 in [13].

We obtained gene- and pathway-based SNP groups by forming the unions of all SNPs within a fixed distance (50 kb) from the coding region of a gene or gene sets, respectively. In collective inference, the overall level of association of each variant group was then inferred by estimating the cross-validation prediction score of disease status (80% of sample individuals were used for inference and prediction was assessed for 20% of individuals) represented by the area under the curve (AUC) of the receiver operating characteristic. The AUC is a measure of prediction performance of classifiers, defined with respect to the receiver operating characteristics, a parametric curve of sensitivity and specificity of predictions obtained by choosing different cutoff values of the statistic used for classification [38]. Its value ranges from ~ 0.5 to 1 with increasing performance, such that values close to 1 imply a capability to predict the case-control status of a new individual based on genotypes with high sensitivity and specificity. Although the AUC is most commonly used as a measure of prediction performance, for our purposes of association testing, it can also serve as a statistic that is free of biases arising from the size of variant sets (number of SNPs contained), the overall composition of gene sets, and other compounding factors that can potentially affect enrichment-based scores [39, 40]. The main advantage of collective inference is the inclusion of interaction effects: the overall AUC scores—optimized with respect to a penalizing parameter so that overfitting is avoided—contain the effect of the aggregated sum of all interactions between variants within the group.

We considered 1580 pathways (with a minimum of 20 SNPs) from the curated Reactome pathway database [41] and used DDA collective loci (CL) analysis to infer their association with T2D disease status (Fig. 1b–e). To convert the AUC values used as statistics into *p*-values (*P*) of the pathway-based variant groups, we selected a subset of SNP groups containing relatively small numbers of SNPs and estimated *p*-values by phenotype-label permutation [30]. The AUC and *P* were highly correlated (Fig. 1c; *r*^2^ = 0.98, quadratic polynomial regression) and led to an estimated Bonferroni threshold (*P* < 3.2×10^− 5^) of AUC > 0.544. The top three pathways were *Binding of TCF/LEF:CTNNB1 to target gene promoters* (*P* = 2×10^− 6^); *Incretin synthesis, secretion, and inactivation* (*P* = 2×10^− 6^); and *Synthesis, secretion, and inactivation of glucagon-like peptide-1 (GLP-1)* (*P* = 5×10^− 6^), whose association levels were comparable to those without interactions. These pathways contain the *TCF7L2* gene, suggesting that this locus largely acts in a monogenic fashion.

**Fig. 2:**
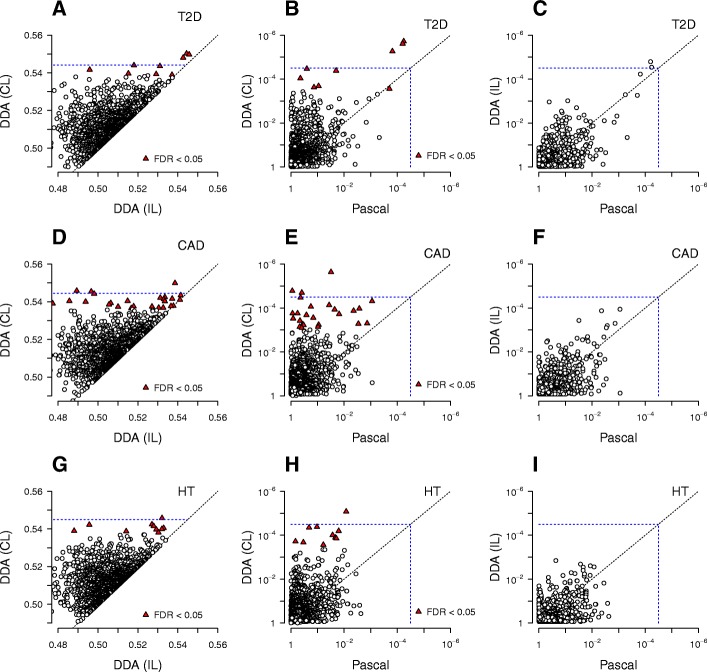
Comparison of inference scores and *p*-values of all pathways in association with T2D (**a**−**c**), CAD (**d**−**f**), and HT (**g**−**i**). The left column compares the DDA score in AUC without (IL) and with interaction effects (CL). The middle and right columns compare *p*-values of pathways from Pascal (mean *p*-value option) with those from DDA CL and IL, respectively. Dotted lines represent the Bonferroni-corrected thresholds

The remaining top-ranked pathways were highly associated with disease status only when interaction effects were included (Fig. 2a). The three *TCF7L2*-related pathways described above exceeded the Bonferroni threshold. We also applied the Benjamini-Hochberg correction [42] to *p*-values of all pathways derived from the AUC, and found six additional pathways with a false discovery rate (FDR) less than 0.05: *Glycerophospholipid biosynthesis*, *Potassium channels*, *Synaptic adhesion-like molecule (SALM) protein interactions at the synapses*, *Peptide ligand-binding receptors*, *Homology-directed repair through homologous recombination or single-strand annealing*, and *Repression of Wnt target genes* (See Additional file 1 for the full list).

The largely monogenic contribution of the *TCF7L2*-proximal region to T2D risk explains the high association of the top three pathways (Fig. 1d and e): GLP-1 is a gut hormone produced by the intestinal L-cells and stimulates insulin secretion from β-cells. The transcription factor TCF4 encoded by *TCF7L2* is a nuclear receptor acting in concert with the Wnt signaling pathway in L-cells to trigger GLP-1 secretion [43]. We also found high associations in pathways involving potassium channels (Fig. 1d), which regulate glucose-induced exocytosis of insulin-containing granules by β-cells [4]. The association of the *SALM protein interactions at the synapses* pathway (*P* = 9.0×10^− 4^) was entirely collective (*P* ~ 1 without interactions). The association of SALM proteins, which regulate synapse formation via interactions with postsynaptic scaffolding proteins [44], with T2D risk has not been reported previously. These pathways were associated with T2D risk via their effects on β-cell insulin secretory dysfunction.

In contrast, the remaining pathways were highly associated with T2D risk (Fig. 1d and e) via their effects on reduced β-cell mass and insulin resistance [2, 3]. The total β-cell mass is plastic over a patient’s lifetime, adjusting via mitogenic division, neogenesis, and apoptosis to allow for responses to changes in load within peripheral tissues (for example, from insulin resistance). Obesity is a major risk factor for insulin resistance, where non-esterified fatty acids (FAs) (*Glycerophospholipid biosynthesis*, Fig. 1d) are believed to play key roles via the inflammatory responses of macrophages (*Immunoregulatory interactions between a lympholid and a non-lymphoid cell*, Fig. 1e) in adipocytes, skeletal muscle, and the liver [45–47]. The *Cell cycle* and *DNA repair* pathways in Fig. 1d, as well as the p53-regulated transcription pathways and *Oncogene-induced senescence* pathway in Fig. 1e, support the roles of β-cell division and apoptosis in T2D risk. In particular, mitochondrial dysfunction contributes significantly to β-cell death [48]: glucose-sensing by β-cells relies on ATP synthesis by mitochondria, which is coupled to closing of the K_ATP_ channel, membrane depolarization, calcium influx, and exocytosis of insulin granules [3]. The associations of intrinsic apoptotic pathways involving mitochondria—*Activation, myristoylation of BH3-interacting domain (BID) and translocation to mitochondria*; *Activation of p53 upregulated modulator of apoptosis (PUMA) and translocation to mitochondria* (Fig. 1e [49, 50])—supports this view of β-cell death as a key contributor to T2D risk. *Unfolded protein response* (Fig. 1d) is integral to endoplasmic reticulum (ER) stress processes implicated in both inflammatory responses leading to insulin resistance and mitochondria-mediated apoptosis of β-cells [51]. We additionally found high association with T2D for *Tachykinin receptor bind tachykinins*, consistent with the finding that substance P-binding neurokinin-1 receptor contributes to insulin resistance in adipocytes [52].

While the level of association with T2D for pathways suggested that the *TCF7L2* locus implicates the GLP-1 and Wnt signaling pathways via independent-SNP effects, the remaining pathways near or immediately below the Bonferroni threshold were mostly associated with T2D via collective effects (Fig. 2a). The latter groups together encompassed a large portion of the suspected pathogenesis mechanisms of T2D. To further assess the increase in association strengths arising from collective effects, we scored the same set of pathways using the recently proposed algorithm Pascal. This method allows for an improved enrichment-based scoring of gene- and pathway-based SNP groups using summary statistics alone [53]. The results were largely similar to those of DDA without interaction effects (Fig. 2b, c), suggesting that collective inference is essential to capture the wide range of T2D risk factors identified (Fig. 1d, e).

**Fig. 3:**
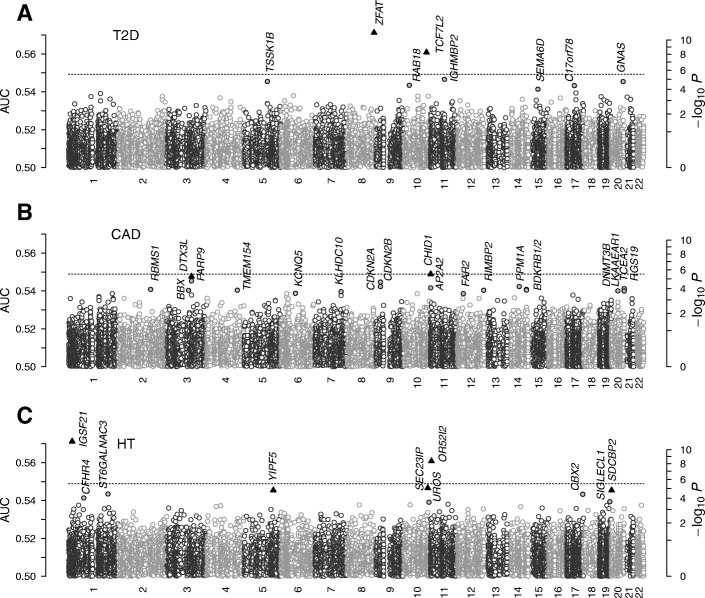
Gene-based scan results under DDA CL for T2D (**a**), CAD (**b**), and HT (**c**). The horizontal lines show the Bonferroni-corrected threshold and the black symbols the genes with an estimated FDR below 0.05

**Fig. 4:**
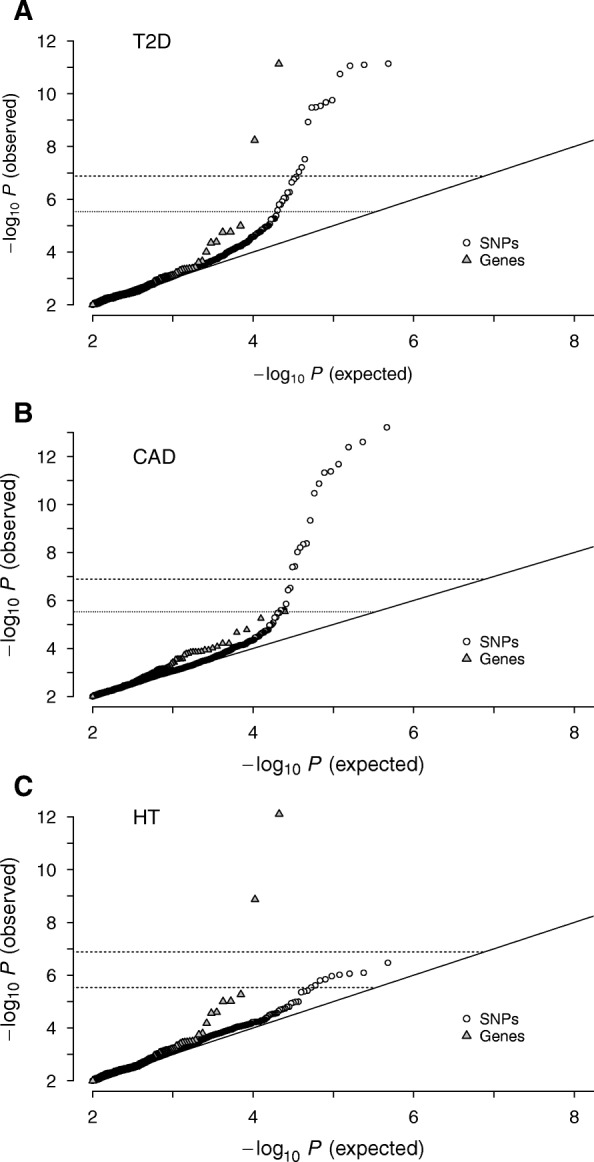
Quantile-quantile plots of independent-SNP and gene-based scores for T2D (**a**), CAD (**b**), and HT (**c**). The horizontal lines represent the Bonferroni-corrected thresholds

We scored the association level of all gene-based SNP groups with T2D analogously (Fig. 3a). Comparing the distribution of the estimated *p*-values of genes with those for independent SNPs with quantile-quantile plots, we found that roughly 10 of the highest-ranked genes showed noticeable deviations from the null distribution (Fig. 4a). The two genes exceeding the Bonferroni-corrected threshold for α < 0.05 were *ZFAT* (*P* = 7×10^− 12^) and *TCF7L2* (*P* = 6×10^− 9^). The latter finding was in line with the top non-interacting SNPs in its locus (Fig. 1a), whereas the association of the former with T2D appeared to be completely collective in nature (IL *P* ~ 1). The *ZFAT* gene encodes a nuclear zinc finger protein essential for maintaining peripheral T cell homeostasis [54, 55]. Adaptive immunity affects T2D risk via inflammatory responses to free FAs in adipocytes [47], and insulin resistance is closely tied to regulatory T cells in adipose tissues [56, 57]. Our finding of the association of *ZFAT* with T2D, which has not been described previously, suggests potential roles of the transcriptional regulation of adaptive immune cells via its effects on insulin resistance. Among other gene products, RAB18, a Ras-related small GTPase involved in vesicle-mediated transport, has been reported to regulate lipid droplets in adipocytes [58].

### Coronary artery disease

**Fig. 5:**
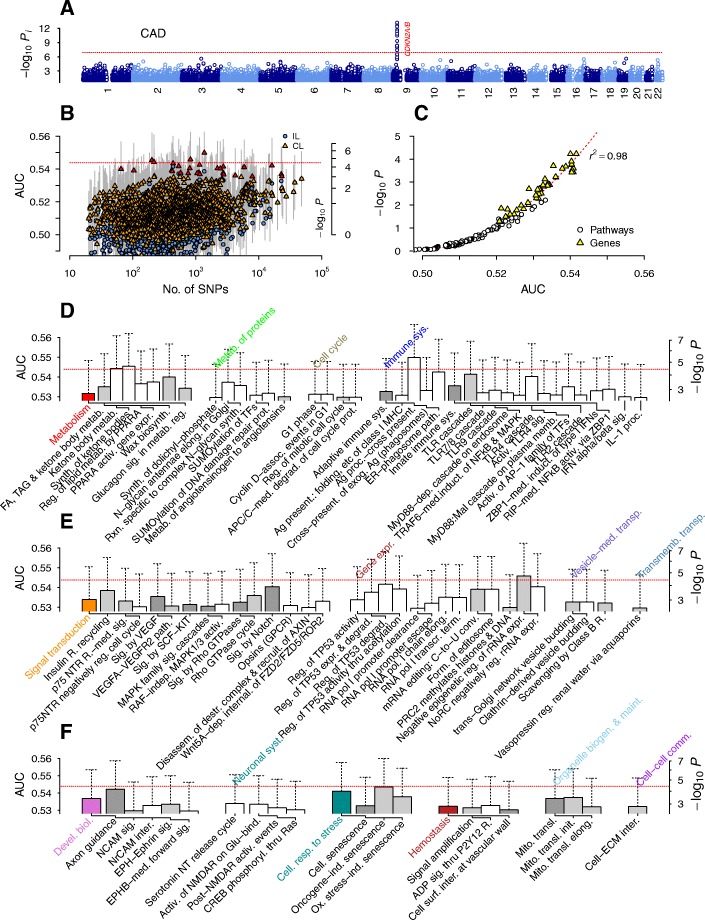
Association test results for coronary artery disease. Horizontal lines represent the Bonferroni threshold. **a** Non-interacting SNPs. **b** Pathways with (CL) and without (IL) interaction effects. Red symbols indicate pathways with an FDR below 0.05. **c** Regression of *p*-values against the AUC. **d**−**f** Top-ranked pathways with AUC > 0.53. Ag., antigen; biogen., biogenesis; biol., biology; cell., cellular; comm., communication; conv., conversion; devel., developmental; ECM, extracellular matrix; ER, endoplasmic reticulum; FA, fatty acid; IFN, interferon; IL, interleukin; maint., maintenance; MHC, major histocompatibility complex; NCAM, neural cell adhesion molecule; NMDAR, n-methyl d-aspartate receptor; ox., oxidative; PPARA, peroxisome proliferator-activated receptor alpha; present., presentation; rxn., reaction; SCF, stem cell factor; surf., surface; TAR, triacylglyceride; TF, transcription factor; TLR, Toll-like receptors; transl., translation; VEGF, vascular endothelial growth factor

The non-interacting SNP *p*-value landscape for CAD (Fig. 5a) was dominated by the *CDKN2A/B* locus on chromosome 9p21, while no other locus showed significant association [8]. The two genes *CDKN2A* and *CDKN2B* encode cyclin-dependent kinase inhibitors regulating the cell cycle, and the associated SNPs lie in enhancer regions known to affect interferon-γ signaling in vascular endothelial cells [59]. However, the direct relevance of this locus in CAD disease mechanisms remains incompletely understood.

The distribution of association levels for pathway-based SNP groups (Fig. 5b) suggested a moderately more pronounced increase in association with the increasing number of SNPs when compared to T2D (Fig. 1b). As in T2D, the regression of collective inference *p*-values on the AUC showed a high correlation (Fig. 5c) and indicated three pathways exceeding the Bonferroni-corrected significance threshold: *Antigen processing and cross presentation* (*P* = 2×10^− 6^), *Negative epigenetic regulation of rRNA expression* (*P* = 1×10^− 5^), and *Synthesis of ketone bodies* (*P* = 2×10^− 5^). There were 30 pathways with FDR < 0.05 (Fig. 5b), of which only three contained *CDKN2A/B* genes (*Cellular responses to stress*, *Oncogene/oxidative stress-induced senescence*, and *Regulation of TP53 degradation*; Additional file 2). As expected, for these three pathways, the association levels were similar with or without interactions, while they were substantially different for ~ 16 of the remaining pathways (Additional file 2), suggesting that they were associated with CAD primarily via non-additive collective interaction effects.

CAD, or atherosclerosis, progresses via a gradual accumulation of apolipoprotein B-containing lipoproteins (LPs) in the extracellular matrix (ECM) beneath the endothelial layer of arterial blood vessels [6]. The inflammatory responses to LPs attract monocytes, which differentiate into macrophages and ingest LP-derived cholesterols to become foam cells. The recruitment of monocytes into the intima involves the concerted action of chemokines and the neuronal axon guidance machinery [60]; we found associations in axon guidance pathways, including ephrin signaling and cell adhesion molecules, in addition to cell-ECM interactions (Fig. 5f). The pathway with the highest association in our results, *Antigen processing and cross presentation*, and its sub-pathway, the *ER-phagosome pathway* (Fig. 5d), describe the uptake, processing, and presentation of exogenous antigens by dendritic cells and other phagocytes via major histocompatibility complex class I molecules [61]. The high association levels of these pathways strongly implicates the uptake of low-density lipoproteins (LDLs) by macrophages via phagocytosis, macropinocytosis, and scavenger receptors, notably the class B receptor CD36 [60, 62] (*Scavenging by class B receptors*, Fig. 5e). The ingested LPs are digested in the lysosome, producing free cholesterols, whose cytosolic accumulation lies at the heart of inflammatory responses leading to macrophage apoptosis. The free cholesterols are re-esterified in the ER and stored as cholesteryl FAs (‘foams’) or transported out into LPs via lipid efflux processes [63]. The pathways in the *Vesicle-mediated transport* group (Fig. 5e) are relevant to these lipid transport processes involving lysosomes and the ER.

In CAD, free cholesterols can also become enriched in the cytoplasmic membrane lipid rafts, triggering pro-inflammatory pattern recognition receptors, notably Toll-like receptors (TLRs) [64–66]. Consistent with this evidence, we found high association levels in a large array of TLR cascade pathways involving myeloid differentiation factor 88 (MyD88), along with other innate immune pathways (Fig. 5d). As expected from the central roles played by FAs in atherosclerosis risks, FA metabolism and related lipid metabolic pathways were also highly ranked (Fig. 5d). In particular, among the highest-ranked was the *Synthesis of ketone bodies* pathway, which produces ketone bodies in the liver from FA oxidation-derived acetyl CoA, which in turn can compensate for glucose-derived molecules in the mitochondrial tricarboxylic acid cycle producing ATP under fasting conditions [67]. These reversible reactions are coupled not only to energy metabolism but also to lipogenesis and cholesterol synthesis. For example, in macrophages, free FAs activate peroxisome proliferator-activated receptor (PPAR) α (*PPARα activates gene expression*, Fig. 5d), which regulates the expression of FA oxidation genes and CD36, generally acting as anti-inflammatory factor [68]. 3-hydroxy-3-methylglutaryl-CoA synthase (HMGCS2), the mitochondrial enzyme involved in ketone body synthesis, is regulated by PPARα, insulin (*Insulin receptor recycling*, Fig. 5e), and glucagon (*Glucagon signaling in metabolic regulation*, Fig. 5d) [67]. The closely related PPARγ inhibits the nuclear factor κB-mediated activation of inflammatory factors via TLR4 signaling, where SUMOylation of PPARγ plays a key role (*SUMOylation of transcription factors*, Fig. 5d) [68]. Ketogenesis and its regulation by PPARs are controlled by sirtuins [67], a class of histone deacetylases regulating diverse aspects of lipid metabolism [69]. Both sirtuins and the nucleolar remodeling complex act via epigenetic control of rRNA expression [70], which explains the high association levels found for *Negative epigenetic regulation of rRNA expression* and transcription pathways involving RNA polymerase I (Fig. 5e).

The overloading of free cholesterol in foam cells can lead to ER stress and cell death by apoptosis (*Cellular responses to stress* in Fig. 5f) regulated by p53 (*Regulation of TP53 degradation* and related pathways, Fig. 5e) during the cell cycle (*Cell cycle* pathways in Fig. 5d). FA oxidation, metabolism, and apoptotic reactions primarily occur in mitochondria, which explains the *Mitochondrial translation* pathways in Fig. 5f. Inflammatory responses of macrophages eventually lead to increased risks for the rupture of fibrous caps separating the intima and lumen, culminating in myocardial infarction (‘stroke’) via thrombosis [60]. It is during this advanced stage of pathogenesis, where risk factors for increased blood pressure, such as the renin-angiotensin-aldosterone system (*Metabolism of angiotensinogen to angiotensins*, Fig. 5d), related signaling pathways (*Signaling by vascular endothelial growth factors*, Fig. 5e), and *Hemostasis* pathways (Fig. 5f), likely exert their effects on atherosclerosis [71].

We used collective inference to score the association levels of all gene-based SNP groups (Fig. 3b). There were two genes near or above the Bonferroni-corrected threshold with an FDR less than 0.05: *CHID1* and *DTX3L*. *CHID1* encodes stabilin-1 interacting chitinase-like protein (SI-CLP) [72], which interacts with stabilin-1, an endocytic scavenger receptor expressed on alternatively activated macrophages capable of LDL uptake [73]. Stabilin-1 shuttles newly synthesized SI-CLP proteins from Golgi compartments to late endosomes for secretion [73]. A recent structural study suggested that SI-CLP possesses saccharide-binding properties [74]. Our previously undescribed finding of the high association level of the *CHID1* gene, along with the presence of scavenger receptor and vesicle-mediated transport pathways in Fig. 5e, suggest potential roles of stabilin-1-mediated secretion of SI-CLP by activated macrophages within an atherosclerotic legion, likely during the stage of monocyte recruitment by endothelial cells, where numerous glycoproteins are involved [60].

*DTX3L* encodes an E3 ubiquitin ligase recently shown to regulate endosomal sorting of the chemokine receptor CXCR4 for lysosomal degradation [75]. One possible source of its association, therefore, is its effect on monocyte recruitment. The third highest ranked gene, *PARP9*, encodes a poly(ADP-ribose) polymerase that acts together with DTX3L in DNA damage response [76, 77] and apoptosis [78]. Together, the high association levels of these two genes suggest their roles within apoptotic foam cells during atherosclerosis. *BDKRB1* and *BDKRB2* encode bradykinin receptors B1 and B2, respectively. These G protein-coupled receptors are central to the regulation of vascular tone and vasoconstriction; infusion of bradykinins binding to B1 and B2 receptors expressed on vascular and smooth muscle cells cause vasodilation [79]. *FAR2* encodes the enzyme fatty acyl-CoA reductase 2, which catalyzes the first step of *Wax biosynthesis* (Fig. 5d) converting FAs into fatty alcohols [80]. Its association with CAD most likely overlaps with that of other FA/ketone body metabolism pathways.

### Hypertension

**Fig. 6:**
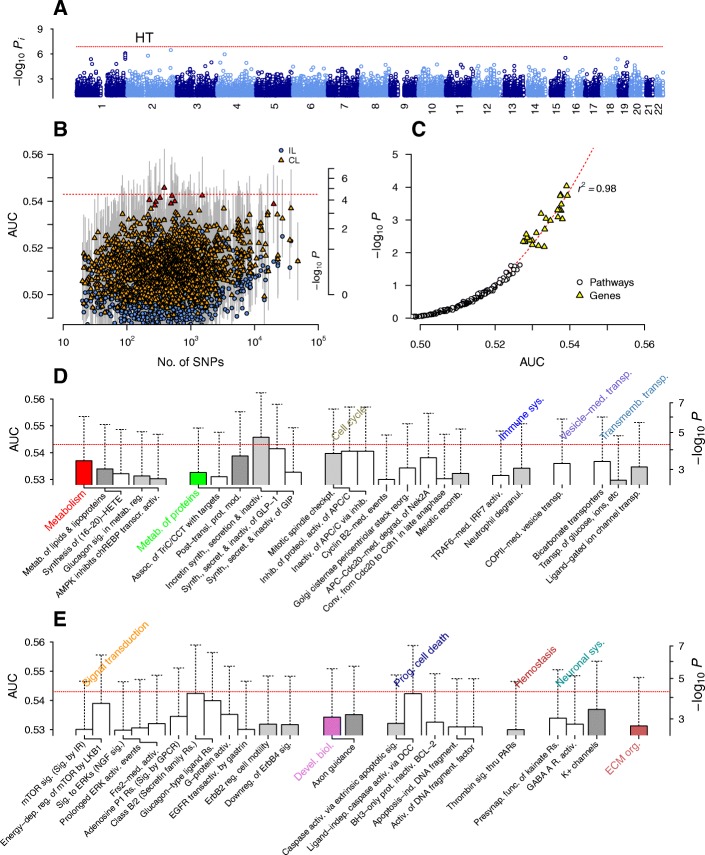
Association test results for hypertension. **a** Non-interacting SNPs. **b** Pathways with (CL) and without (IL) interaction effects. Red symbols indicate pathways with an FDR below 0.05. **c** Regression of *p*-values against the AUC. **d**, **e** Top-ranked pathways with AUC > 0.53. 5-HETE, 5-hydroxy-eicosatetraenoic acid; AMPK, AMP-activated kinase; assoc., association; checkpt., checkpoint; conv., conversion; COPII, coat protein 2; degranul., degranulation; downreg., downregulation; EGFR, epidermal growth factor receptor; fragment., fragmentation; func., function; GABA, gamma-aminobutyric acid; GIP, glucose-dependent insulinotropic polypeptide; indep., independent; inhib., inhibition; mod., modification; mTOR, target of rapamycin; org., organization; presynap., presynaptic; proteol., proteolytic; recomb., recombination; reorg., reorganization; transactiv., transactivation; TRiC/CCT, TCP1-ring complex or chaperonin containing TCP1

The IL profile of levels of association with HT did not show any genome-wide significant loci (Fig. 6a) [8]. In contrast, under collective inference, we found three pathways above or at the Bonferroni-corrected threshold [*Incretin synthesis, secretion, and inactivation*, *P* = 8×10^−6^; *Class B/2 (secretin family receptors)*, *P* = 4×10^−5^; *Ligand-independent caspase activation* via *DCC*, *P* = 5×10^−5^] and 11 with an FDR less than 0.05 (Fig. 6b; see Additional file 3 for the full list). Arterial blood pressure is determined by the product of two main factors, blood flow and resistance (Ohm’s law) [7], which are sensitive to overall blood volume and vascular tone, respectively. Both aspects of HT risk pathogenesis are affected by obesity [81]. In particular, the high association level of the incretin metabolism pathway group with HT (Fig. 6d) parallels that with T2D (Fig. 1d) and is consistent with extensive evidence for the involvement of the incretin system in cardiovascular disease pathogenesis [82]. Notably, the lack of monogenic loci in Fig. 6a suggested that the high association level of incretin pathways with HT is collective in nature, in contrast to that with T2D where the *TCF7L2* locus made the dominant contribution (Fig. 1a). Insulin resistance in peripheral adipocytes contributes to HT risk under obesity, which explains the association of lipid metabolism/signaling pathways with HT in Fig. 6d [*Synthesis of (16–20)- hydroxyeicosatetraenoic acids (HETE)*, *Glucagon signaling in metabolic regulation*, and *AMP-activated protein kinase inhibits carbohydrate response element binding protein (ChREBP) transcription activity*] and that of the *Glucagon-type ligand receptors* pathway in Fig. 6e. ChREBP is a key transcriptional regulator of lipid metabolism in the liver associated with obesity and T2D [83].

The association of *Cell cycle*, apoptosis pathways, *Axon guidance*, and the hemostasis pathway (*Thrombin signaling through proteinase-activated receptors*) with HT (Fig. 6d and e) parallels that with CAD (Fig. 5). Together with *ECM organization* (Fig. 6e), these associations suggest increased HT risks via elevated vascular resistance from atherosclerotic lesions.

We also found high association levels for *Transmembrane transport of small molecules* pathways (Fig. 6d), including *Bicarbonate transporters* and *Ligand-gated ion channel transport*. Bicarbonate, a major waste product of mitochondrial respiration, must be disposed of to maintain body pH, and affects cardiovascular functions via pH imbalance in the heart [84]. Dysfunctions of renal filtering of salts can result in abnormal retention of sodium and cause osmotic expansion of blood volume [7]. Neuronal system pathways (*Presynaptic function of kainate receptors*, *Potassium channels*; Fig. 6e), on the other hand, suggest the relevance of the sympathetic nervous system controlling vasoconstriction.

In comparison to IL, ~ 10 gene-based groups showed substantially stronger deviations from the null distribution (Fig. 4c) with two genes significantly exceeding the Bonferroni-corrected threshold for HT: *IGSF21* (*P* = 8×10^−13^) and *OR52I2* (*P* = 1×10^−9^).

### Type 2 diabetes in mice

**Fig. 7:**
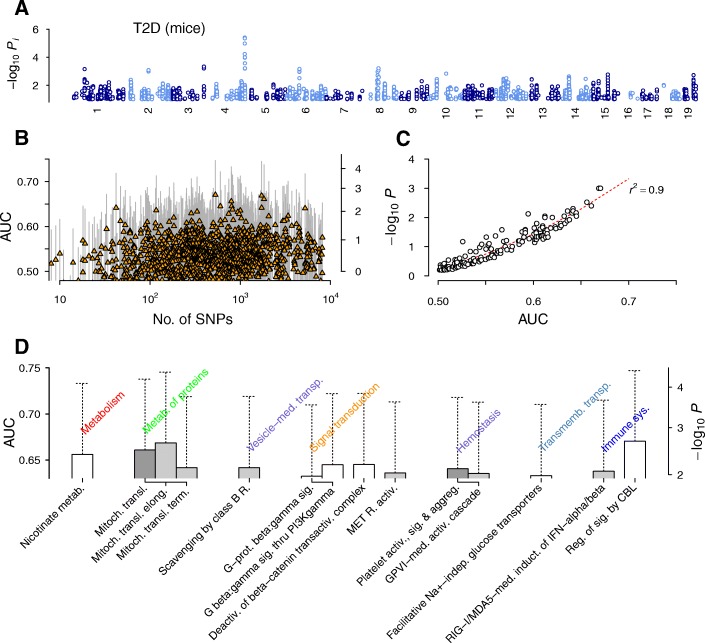
Association test results for type 2 diabetes in mice. **a** Non-interacting SNPs. **b** Distribution of pathway scores with interaction effects. **c** Regression of *p*-values against the AUC. **d** Top-ranked pathways with AUC > 0.63. Aggreg., aggregation; GPVI, glycoprotein VI; induct., induction; MDA5, melanoma differentiation-associated protein 5; mitoch., mitochondrial; RIG-I, retinoic acid-inducible gene I

To further support our results, we analyzed an independent data set reflecting a T2D phenotype in mice. We used a recent genome-wide data set of outbred mice by Nicod et al. [34], who characterized the animals with a comprehensive list of physiological and behavioral traits. Using a glucose level cutoff typical for diabetic mice [85], we formed a case-control data set (61 case and 1131 control animals). The genome-wide IL association levels of SNPs were low and no variants exceeded the Bonferroni-corrected threshold (Fig. 7a), reflecting the smaller sample size of the mouse data set compared to the human data sets. Although the overall range of AUC values for pathways from the mouse data (Fig. 7b) was higher than that of their human counterparts (Figs. 1b, 5b, and 6b), *p*-value estimation revealed that the absolute significance levels of highly ranked pathways were lower (*P* > 10^−3^, Fig. 7b and c) as expected from the smaller sample size.

Nevertheless, the top-ranked pathways (Fig. 7d) were highly concordant with T2D pathogenesis and complemented the analysis outcome of human data (Fig. 1d and e): among the top-ranked pathways, we found *Deactivation of beta-catenin transactivation complex* of Wnt signaling, *Mitochondrial translation* pathways, and the *Facilitative Na*^*+*^*-independent glucose transporters* pathway (Fig. 7d). The appearance of the Wnt signaling pathway is consistent with its strong association in human T2D (Fig. 1e), where the role of mitochondrial translation in apoptotic β-cell death has also been noted (programmed *cell death* pathways in Fig. 1e).

## Discussion

Together, our analyses of data sets for three metabolic diseases (T2D, CAD, and HT; Figs. 1, 5, 6) demonstrate that collective inference, which incorporates the cumulative sum of non-additive interaction effects involving variants within a pathway group, can reveal novel associations not detectable by IL methods. Notably, these associations arose from data sets of relatively small sizes for which IL yielded few (T2D and CAD) or no (HT) genome-wide significant loci. The absolute significance levels of top-ranked pathways generally were near or below the Bonferroni-corrected thresholds, which are substantially more conservative for pathways than for SNPs because most pathways are hierarchically related. In a different meta-analysis of psychiatric disorders using DDA [33], we performed down-sampling to find evidence that the absolute association levels of the top-ranked pathways become comparable to those obtained with the Bonferroni-corrected threshold under sample sizes of around several thousands. The significance levels we observed in this study (Figs. 1, 5, 6) are therefore consistent with this trend under the current sample sizes (*n* ~ 5000; case/control combined).

Although recent large-scale meta-analyses have achieved high power with combined sample sizes of *n* ~ 10^4^ or more, interpretations of IL analysis face the difficulty of assigning causal genes from which the lead SNP (or its correlated partner in LD) derives its effect, making the biological interpretation of established loci ambiguous. Existing gene- and pathway-based scoring methods that combine IL *p*-values into statistical significance scores for each variant group [39] overcome this difficulty, but with the use of minimum or geometric mean of IL *p*-values for each group, lack the means to account for interaction effects. At the expense of requiring individual-level genotype data instead of summary statistics, our approach provides association scores of variant groups while including collective interaction effects via the AUC, a measure of disease status prediction evaluated by cross-validation. As shown in Figs. 1c, 5c, and 6c, this measure is in general highly correlated with the *p*-values of the variant groups, and suggests that the significance threshold of a pathway (*P* < 10^− 5^) is achieved much earlier than prediction fidelity (AUC > 0.7) with increasing power. By comparing DDA with the existing pathway-scoring approach Pascal utilizing summary statistics only (Fig. 2), we conclude that the performance of DDA without interaction effects (IL) is similar to those of other methods, whereas DDA collective inference discovers many hidden associations for which interaction effects play dominant roles.

For all three of the metabolic disease data sets considered, the top-ranked pathways near or below the Bonferroni-corrected thresholds together covered large parts of known or suspected disease mechanisms, suggesting their biological relevance. For T2D (Fig. 1d and e), they comprised (monogenic) GLP-1-related pathways stimulating secretion of insulin-containing granules by β-cells, potassium channels regulating glucose-induced action potential firing, and the group of pathways underlying stress-induced β-cell death, including unfolded protein response, cell cycle/DNA repair regulated by p53, and mitochondrial apoptosis pathways. For CAD, the pathways covered different stages of atherosclerosis progression (Fig. 5d–f); recruitment of circulating monocytes by ECM interactions and axon guidance pathways; lipoprotein uptake by macrophages via phagocytosis; inflammatory response via scavenger receptors, TLR, and cross-presentation pathways; macrophage apoptosis via cellular stress; and thrombosis of ruptured atherosclerotic lesions. Elements of T2D (GLP-1 signaling, lipid metabolism) and CAD risk factors (cell cycle, axon guidance, ECM organization, apoptosis, and hemostasis) were present in HT (Fig. 6d-e), in addition to transmembrane transport pathways related to salt retention. Many developmental pathways we observed in the CAD outcome may affect disease risk via indirect means, such as transcription factor expression regulation by miRNAs [28, 86].

Although gene-based scoring revealed relatively fewer highly associated groups than pathways, it suggested novel genes potentially implicated in pathogenesis not found in IL analyses, notably for CAD (Fig. 3). Overall, the increased level of association found for pathways in comparison to genes under relatively smaller sample sizes is consistent with our previous observation in psychiatric disorders [33], and suggests that polygenicity of common diseases is better captured by gene sets than by individual genes.

The T2D-associated pathways (β-catenin/Wnt signaling and mitochondrial translation) from the outbred mice suggest that our inference algorithm produces consistent results not only over different populations (humans versus mice) and phenotype classes (T2D diagnosis versus glucose levels), but also under both high and low power with disparate sample sizes (~ 1000 versus less than 100 case individuals).

## Conclusions

We analyzed lipid-metabolic and inflammatory pathways underlying common metabolic diseases, using an algorithm that takes into account large-scale epistatic effects. This analysis approach enabled us to discover SNP groups whose association was primarily many-bodied in nature. These SNP groups consisted of arrays of pathways previously thought to be linked to metabolic diseases. Thus, pathway-based testing approaches incorporating large-scale interaction effects can reveal hidden association effects, using samples sizes much smaller than those needed to achieve similar levels of statistical significance in single-SNP-based methods.

## Additional files

Additional file 1: List of top-ranked pathways associated with type 1 diabetes. (XLSX 20 kb)

Additional file 2: List of top-ranked pathways associated with coronary artery disease. (XLSX 21 kb)

Additional file 3: List of top-ranked pathways associated with hypertension. (XLSX 19 kb)

## Abbreviations

AUC: Area under the curve
CAD: Coronary artery disease
ChrREBP: Carbohydrate response element binding protein
CL: Collective loci
DDA: Discrete discriminant analysis
ECM: Extracellular matrix
ER: Endoplasmic reticulum
FA: Fatty acid
FDR: False discovery rate
HT: Hypertension
IL: Independent loci
LD: Linkage disequilibrium
LDL: Low-density lipoprotein
LP: Lipoprotein
PPAR: Peroxisome proliferator-activated receptor
SALM: Synaptic adhesion-like molecules
SI-CLP: Stabilin-1 interacting chitinase-like protein
SNP: Single nucleotide polymorphism
T2D: Type 2 diabetes
TLR: Toll-like receptor
WTCCC: Wellcome Trust Case-Control Consortium
